# Odd–even effect for efficient bioreactions of chiral alcohols and boosted stability of the enzyme†

**DOI:** 10.1039/d0ra05406b

**Published:** 2020-07-29

**Authors:** Mark Bülow, Alexa Schmitz, Termeh Mahmoudi, Dana Schmidt, Fabian Junglas, Christoph Janiak, Christoph Held

**Affiliations:** Laboratory of Thermodynamics, Technical University Dortmund 44227 Dortmund Germany christoph.held@tu-dortmund.de; Institut für Anorganische Chemie und Strukturchemie, Heinrich-Heine-Universität Düsseldorf 40204 Düsseldorf Germany

## Abstract

We describe a holistic approach for achieving a nearly quantitative conversion for an enzymatic reaction while simultaneously increasing the long-term stability of the enzyme. The approach provided chemical control of bioreactions by utilizing newly synthesized tetrahydrothiophene-based ionic liquids (THT ILs). We showcased its power by using THT-ILs as additives at a low concentration (only 10 mmol L^−1^) in the alcohol dehydrogenase (ADH)-catalyzed synthesis of methylated 1-phenylethanol (Me-PE). We discovered an “odd–even” effect of the IL-cation chain length: Me-PE displayed beneficial interactions with THT ILs having odd-numbered chain lengths and deleterious interactions with those having even-numbered chain lengths. An intermolecular thermodynamic simulation of the bulk phase and critical micelle concentration investigations of the local surroundings of the THT-ILs proved the occurrence of these interactions, and these two methods confirmed the odd–even effect from different perspectives. Additionally, storing the ADH enzyme in pure THT IL at room temperature allowed for a boosted long-term stability of the enzyme (500 times greater than that in aqueous buffer) without the need for freezing.

## Abstract 2

We describe a holistic approach for achieving a nearly quantitative conversion for an enzymatic reaction while simultaneously increasing the long-term stability of the enzyme.

## Graphical abstract

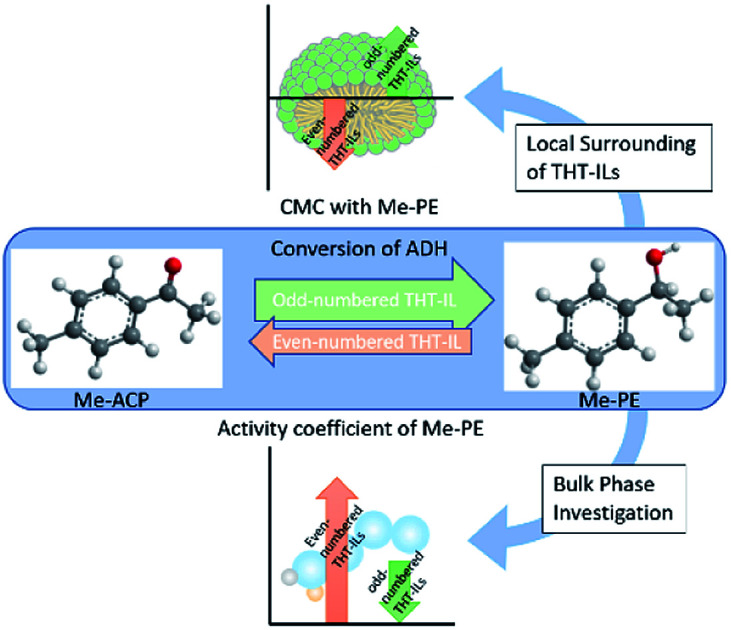

There is currently a demand to boost the selectivity and conversion for the reactions of bulk chemicals to fine chemicals. It is known that reactions can be controlled *via* careful choice of the additives.^1,2^ The benefit of such additives is to increase reactant affinity towards the solvent and to help overcome solubility issues of the reactant. Additives can increase chemoselectivity by discriminating against improper functional groups.^1^ Additional spatial control is induced by the formation of micelles. Micelles are often applied to reactions involving transitions from organic solvents to aqueous media, forming surface-stable colloidal dispersions with water-insoluble reactants or products.^3^ In these colloidal dispersions, micelles have been used to concentrate the reactants within their centers, *i.e.*, to locally stabilize the enzyme, reactants, or products.^4,5^ Using micelles can thus allow control of the reaction rate, reaction mechanism, and regioselectivity, as well as stereoselectivity, even for bioreactions.

For enzyme-catalyzed reactions, selectivity is usually not an issue. Of greater concern is the degradation of the enzyme connected to its stability and activity, as well as the need for a bifunctional solvent that satisfies the needs of, on the one hand, maintaining the high stability of the enzyme (aqueous media) and, on the other hand, providing an environment yielding sufficient solubility of hydrophobic reactants.^6^ Thus, the selection of co-solvent additives involves multidimensional optimization to maximize reactant solubility, stability and activity of the enzyme, and overall conversion and kinetics.

This article focuses on a holistic approach to control enzyme-catalyzed reactions with co-solvents—to improve solubility, conversion and activity without losing specifics such as high enantioselectivity. The co-solvents used in this work were nonvolatile ionic liquids (ILs), known to possibly benefit conversion.^7,8^ As (co-)solvents in (bio)reactions,^9^ ILs have already been used to control the hydrophobicity of the reaction media, with this control effected by choosing the appropriate IL-anion, IL-cation alkyl chain length, polarity, and viscosity.^10,11^ The influence of the IL on the equilibrium conversion was proposed to be due to favorable interactions made by the IL both with reactants and with products.^10,12,13^

In this work, tetrahydrothiophene-based ILs (THT-ILs, Scheme 1, details in ESI†) were introduced as co-solvents to bioreactions. IL-cations made of cyclic sulfonium were used to weaken the electrostatic forces with the IL-anion, allowing a higher availability of the IL-ions and a characteristically low viscosity.^14^

**Scheme 1:**
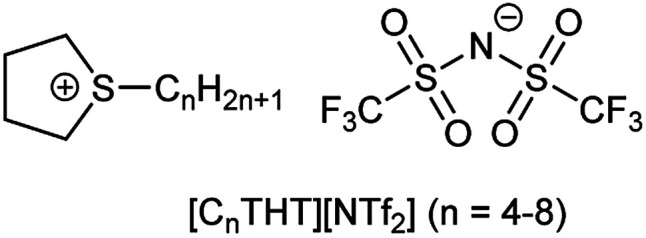
The chemical structures of the tetrahydrothiophenium ([C_*n*_THT]^+^) cation and bis(trifluoromethanesulfonyl)imide ([NTf_2_]^−^) anion.

A holistic study of the influence of THT-ILs was performed with the alcohol dehydrogenase (ADH) reaction at room temperature. In the ADH reaction that was carried out, 4-methylacetophenone (Me-ACP) was reacted with 1-(4-methylphenyl)ethanol (Me-PE) as depicted in Scheme 2. ADH270 was used as the enzyme and the reaction involved consumption of the co-factor nicotinamide adenine dinucleotide (NADH).

**Scheme 2:**
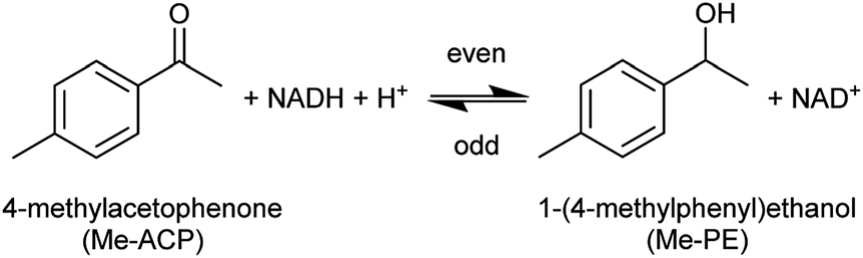
Reaction scheme for the alcohol-dehydrogenase-catalyzed reaction of Me-ACP to Me-PE with the mandatory co-factor NADH.

Maintaining a suitable stability of an enzyme in the presence of an IL is usually a challenge.^15^ Accordingly, as a first step, the influence of the pure THT-IL on the ADH activity was investigated over a time span of 31 days. For comparison, the stability of ADH in an aqueous buffer solution was tested, and here the ADH was found to be active only for 2 h at room temperature. In pure [C_5_THT][NTf_2_], it was active about 500 times as long (*cf.* Fig. S4, ESI†). Due to its impeding of enzyme degradation, the THT-ILs were found to be generally suitable for enzyme reactions.

The influence of THT-ILs on the percent conversion of the ADH reaction was monitored over time by using UV/Vis spectroscopy at *λ* = 340 nm to measure the (decreasing) concentration of NADH. At this wavelength, the ILs did not perturb the UV signal (Fig. S1, ESI†). The equilibrium conversion was reached when the extinction at *λ* = 340 nm approached a steady level (Fig. S2 and Table S6, ESI†), as reported in previous publications.^16^ To allow for an assessment of the impact of IL on the conversion, the IL-anion ([NTf_2_]^−^) was fixed and the overall IL concentration in the mixture was held constant at 10 mmol per liter of aqueous buffered solvent. Reactants and enzyme were added to the aqueous buffer solution at a constant temperature (see Table S5, ESI† for starting concentrations). The only variable between the experiments was the alkyl chain length of the IL-cation (C_4_–C_8_). All experiments were performed at 25 °C, 1 bar and pH 7. Experimental percent conversion for the reaction in the neat aqueous buffer was 87% (base line in Fig. 1).

**Fig. 1:**
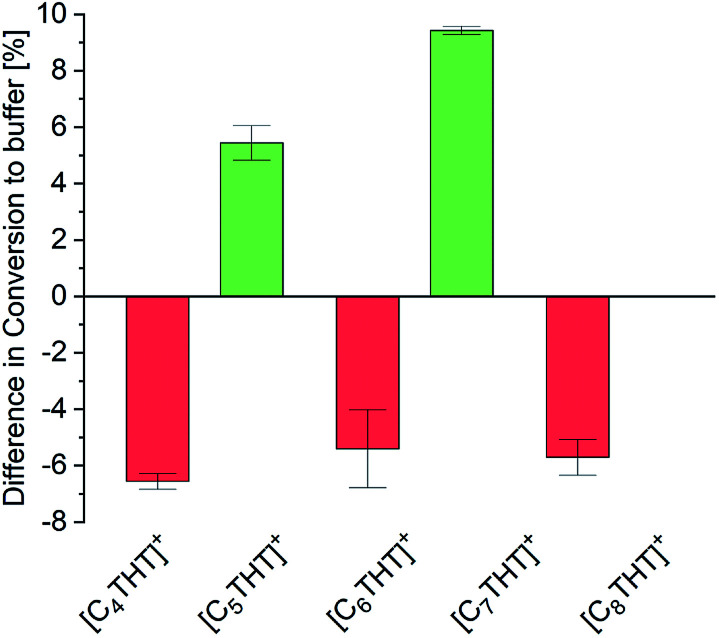
Differences of the experimental percent conversion of the ADH reaction between that using buffer + IL and that using neat buffer (base line) for the various [C_*n*_THT]^+^-cations tested.

The percentage conversion of ADH-catalyzed reactions has been reported to decrease upon addition of the IL 1-butyl-3-methylimidazolium-[NTf_2_], [C_4_mim][NTf_2_], *i.e.*, compared to that of pure buffer solution.^8^ In the presence of THT-ILs with even-numbered chain lengths, that is, with [C_4_-, C_6_- and C_8_-THT]^+^, comparable negative effects on the conversion were observed (Fig. 1). Thus, the percent conversion of the reaction in neat buffer was higher than those of the reactions including 10 mmol L^−1^ of these ILs. Please note that increasing the concentrations of these even-numbered-chain-length ILs to higher than 10 mmol L^−1^ did not improve the percent conversion (data not shown).

As seen in Fig. 1, two different effects of the odd-numbered IL-cation chain length on the percent conversion of the ADH reaction were observed. First, the longer the alkyl chain of the IL-cation, the higher the percent conversion. This behavior was probably caused by the increased hydrophobic character of the IL with an increased chain length. The increased hydrophobicity allowed for a greater solubility of the reactant in the buffer + IL solution than in neat buffer. Second and counterintuitively, ILs with odd-numbered THT-cations were observed to be highly beneficial for the conversion. Addition of only 10 mmol L^−1^ IL to the aqueous buffer led to an increased conversion, *i.e.*, compared to the reaction in neat buffer, with the inclusions of [C_5_THT][NTf_2_] and [C_7_THT][NTf_2_] yielding percent conversions of 92% and greater than 96%, respectively. In contrast to the THT-ILs with even-numbered IL-cation chain lengths, those with the odd-numbered ones shifted the reaction equilibrium by about 10% to reach an almost quantitative conversion. The observed effects were not found for nonmethylated ACP and 1-PE (data not shown), indicating the effect to be due to interactions between THT-ILs and Me-PE/Me-ACP.

Such “odd–even” effects have been mainly described for pure components,^16–20^*e.g.*, involving shifts in melting points for alkanes. And these effects have also been described in the literature for multi-component systems,^17,18^ such as for partition coefficients of a salt in ternary systems including ILs.^19^ However, to the best of our knowledge, odd–even effects on a catalytic reaction have not been reported in the literature so far.

For achieving qualitative explanations from an intermolecular viewpoint, the bulk phase in the equilibrated reaction system was investigated by means of the electrolyte perturbed-chain statistical associating fluid theory (ePC-SAFT) equation of state. ePC-SAFT has already been successfully used to predict the influence of additives, as well as of ILs, on reaction equilibria of enzyme-catalyzed reactions with reasonable accuracy.^8,13,20^ Intermolecular forces, like van der Waals or H-bonding forces, are specifically accounted for. Details of the ePC-SAFT calculations performed in the current work are listed in the ESI. And the required parameters are given in Tables S2 and S3;† these parameters were fitted to IL densities (Table S4†). A key property here is the activity coefficient: the greater the activity coefficient of a compound in a given medium, the greater the repulsive forces between this compound and the medium. ePC-SAFT-predicted activity coefficients at equilibrium conditions of the reaction *γ*^eq^_i_ (in this work i = Me-ACP (orange bars) or Me-PE (blue bars)) are shown in Fig. 2.

**Fig. 2:**
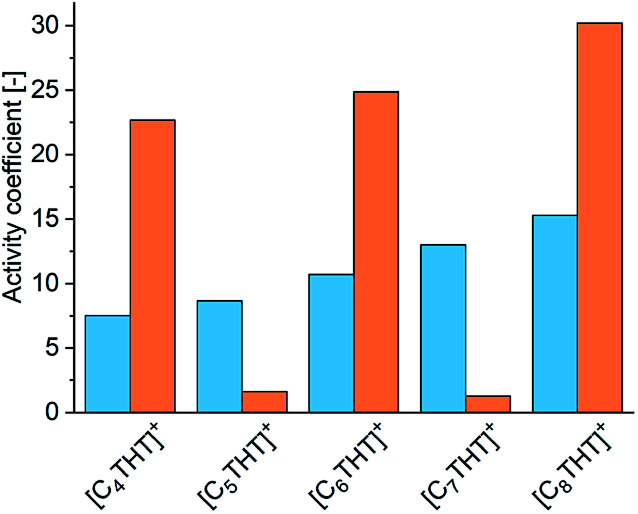
Activity coefficients of the reactant Me-ACP (blue bars) and the product Me-PE (orange bars) in the reaction medium in the presence of 10 mmol L^−1^ THT-IL with various alkyl chain lengths of the [C_*n*_THT]^+^-cation.

Two effects of the THT-IL on the activity coefficients were deduced. First, *γ*^eq^_Me-ACP_ (blue bars in Fig. 2) was observed to rise monotonically with increasing length of the IL-cation chain, indicating increasing repulsive forces between Me-ACP and the reaction medium. An odd–even effect was not observed for the reactant. In contrast, *γ*^eq^_Me-PE_ showed the alternation, *i.e.*, the same odd–even effect as observed on the percent conversion of the reaction illustrated in Fig. 1. Drastically lower *γ*^eq^_Me-PE_ values were observed for the THT-ILs with the odd-numbered chain lengths than for those with the even-numbered ones, indicative of stronger attractive forces of Me-PE with the reaction medium for the odd-numbered cases; this feature allowed a shift in equilibrium towards the product side.^10^ The theoretical studies of the bulk-phase effects of the THT-ILs qualitatively matched the experimental observation on the reaction equilibrium. However, this match did not exclude the occurrence of additional spatial effects. ePC-SAFT does not allow to cover local-composition effects in the surrounding of the THT-ILs.

For further insight into local effects, the critical micelle concentrations (CMCs) of the THT-ILs were measured (Tables S7, S8 and Fig. S3 in the ESI†). Reactions taking place near or above the CMC frequently exhibit increased percent conversions, due to a locally increased solubility of the reactant or/and catalyst in the micelles.^5^ Also, inhibited interactions with the aqueous solution in CMC-supported organic reactions hamper the decomposition of reactants.^21^ Bioreactions most commonly take place in aqueous media, and CMC effects on such catalytic reactions have not yet been investigated in detail. Nevertheless, bioreactions are often limited by issues regarding the stability of the catalyst or solubility levels of the reactants. To understand the interactions in the surrounding of the THT-ILs, the CMCs were measured in pure water as a reference and in the presence of the reaction product Me-PE—with the CMC defined as the value above which the micelles formed (Table S8, ESI†). The results are illustrated in Fig. 3 as the difference for each IL between the CMC of the IL in Me-PE + water and that in pure water (*i.e.*, without Me-PE). The CMC in pure water was found to be close to 10 mmol L^−1^, *i.e.*, the IL concentration that was used. Me-PE then perturbed (favored) the micellar formation of odd-numbered (even-numbered) THT-ILs, hence causing an odd–even effect of the CMC induced by Me-PE.

**Fig. 3:**
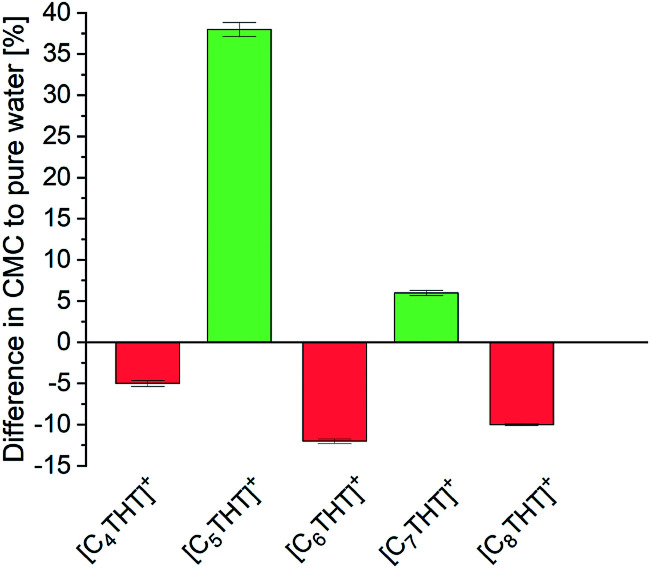
Difference, for each [C_*n*_THT]-IL tested, between the CMC of the IL in water + Me-PE and that in pure water (baseline).

This alternation observed corresponded to the observed odd–even effects on the percent conversion of the ADH reaction (*cf.*Fig. 1). Me-PE prohibited IL–micelle formation due to increased interactions with odd-numbered THT-ILs (*cf.* activity coefficient), which benefited the conversion. In contrast, micelle formation for the even-numbered THT-ILs was shifted towards lower IL concentrations caused by interactions with Me-PE; this feature impeded the ADH reaction. Thus, the enhanced conversion can be considered to be “self-induced” by Me-PE in the presence of THT-ILs with IL-cations with odd-numbered chain lengths. The effect of increasing solubility levels of the reactants/catalysts in micelles described in the literature^5^ was obviously not the reason behind the findings, as local solubilities of reactants/catalysts were ensured in the presence of the very diluted aqueous reactant solutions (*c*_Me-ACP_ < 1 mmol L^−1^) used in this work (see Table S5 in ESI†). This solubility effect might become more important for increasing reactant concentrations that require increased enzyme concentrations in turn.

In conclusion, the holistic approach covers the design of the control of ADH reactions *via* the addition of specially designed THT-ILs. First, the THT-ILs were shown to be capable of drastically enhancing the temporal activity of the ADH enzyme for at least five weeks even at room temperature. Second, a never-before-reported “self-induced” odd–even effect was taken advantage of to enhance the reaction to a nearly quantitative conversion when applying only a low concentration of 10 mmol L^−1^ of the THT-ILs. This effect was traced to the molecular interaction of the methylated product Me-PE with the odd-numbered [C_*n*_THT]^+^-cation. CMC measurements revealed corresponding odd–even effects, inhibiting the formation of micelles for odd-numbered THT-ILs due to the formation of strong interactions with the product Me-PE. The holistic approach presented here, involving IL tuning to take advantage of the new odd–even effects for novel THT-ILs, has the potential for process intensification by effecting a spatial–temporal control over the important reaction parameters.

## Conflicts of interest

There are no conflicts to declare.

## Supplementary Material

RA-010-D0RA05406B-s001

## References

[cit1] Collins K. D., Glorius F. (2013). Nat. Chem..

[cit2] Churcher I. (2013). Nat. Chem..

[cit3] Cordes E. H., Dunlap R. B. (1969). Acc. Chem. Res..

[cit4] Dwars T., Paetzold E., Oehme G. (2005). Angew. Chem., Int. Ed..

[cit5] La Sorella G., Strukul G., Scarso A. (2015). Green Chem..

[cit6] Koeller K. M., Wong C. H. (2001). Nature.

[cit7] (a) Holbrey J. D. , Turner M. B. and Rogers R. D., in Ionic Liquids as Green Solvents, ed. R. D. Rogers and K. R. Seddon, American Chemical Society, Washington, DC, 2003, vol. 856, pp. 2–12

[cit8] Wasserscheid P. and Welton T., Ionic liquids in synthesis, John Wiley & Sons, 2008

[cit9] Kragl U., Eckstein M., Kraftzik N. (2002). Curr. Opin. Biotechnol..

[cit10] Voges M., Fischer C., Wolff D., Held C. (2017). Org. Process Res. Dev..

[cit11] Kaftzik N., Wasserscheid P., Kragl U. (2002). Org. Process Res. Dev..

[cit12] Held C., Sadowski G. (2016). Annu. Rev. Chem. Biomol. Eng..

[cit13] Wangler A., Canales R., Held C., Luong T. Q., Winter R., Zaitsau D. H., Verevkin S. P., Sadowski G. (2018). Phys. Chem. Chem. Phys..

[cit14] Guo L., Pan X., Zhang C., Wang M., Cai M., Fang X., Dai S. (2011). J. Mol. Liq..

[cit15] Madeira Lau R., Sorgedrager M. J., Carrea G., van Rantwijk F., Secundo F., Sheldon R. A. (2004). Green Chem..

[cit16] Voges M., Abu R., Gundersen M. T., Held C., Woodley J. M., Sadowski G. (2017). Org. Process Res. Dev..

[cit17] Marčelja S. (1974). J. Chem. Phys..

[cit18] Uchida H., Miyata K., Oba M., Ishii T., Suma T., Itaka K., Nishiyama N., Kataoka K. (2011). J. Am. Chem. Soc..

[cit19] Belchior D. C. V., Sintra T. E., Carvalho P. J., Soromenho M. R. C., Esperança J. M. S. S., Ventura S. P. M., Rogers R. D., Coutinho J. A. P., Freire M. G. (2018). J. Chem. Phys..

[cit20] Wangler A., Loll R., Greinert T., Sadowski G., Held C. (2019). J. Chem. Thermodyn..

[cit21] Bica K., Gärtner P., Gritsch P. J., Ressmann A. K., Schröder C., Zirbs R. (2012). Chem. Commun..

