# Supplementary material for: Odd–even effect for efficient bioreactions of chiral alcohols and boosted stability of the enzyme
Source: RSC Adv. 2020 Jul 29;10(47):28351–4. doi: 10.1039/d0ra05406b (PMC9055634; doi:10.1039/d0ra05406b)
Supplement: RA-010-D0RA05406B-s001 [file RA-010-D0RA05406B-s001.pdf]

Electronic Supplementary Material (ESI) for RSC Advances.
This journal is © The Royal Society of Chemistry 2020

SUPPORTING INFORMATION

Odd-even effect for efficient bioreaction of chiral alcohols and boosted stability of the enzyme

Mark Bülow,[†,‡] Alexa Schmitz,[#,‡] Termeh Mahmoudi,[†] Dana Schmidt,[#] Fabian Junglas,[#] Christoph Janiak,[#] and Christoph Held[†*]

AUTHOR ADDRESS.

[†] Laboratory of Thermodynamics, Technical University Dortmund, Emil-Figge-Str 70, 44227 Dortmund, Germany. Fax: +49-231-755-2075; Tel: +49-231-755-2086 E-mail: christoph.held@tu-dortmund.de

[#] Institut für Anorganische Chemie und Strukturchemie, Heinrich-Heine-University Düsseldorf, 40204 Düsseldorf, Germany. Fax: +49-211-81-12287; Tel: +49-211-81-12286. E-mail: janiak@uni-duesseldorf.de

Contents

1. Abbreviations 2
2. Syntheses of tetrahydrothiophene-ILs 2
3. Thermodynamics of reaction equilibria 3
4. ePC-SAFT modelling 4
5. Density measurements for THT-ILs 6
6. Reaction-equilibrium measurements 7
7. Measuring the critical micelle concentration 8
8. ADH Reaction in neat THT-ILs 10
9. References 11

ABBREVIATIONS

ACP, acetophenone

ADH, alcohol dehydrogenase

CMC, critical micelle concentration

$[C_4mim]^+$, 1-butyl-3-methylimidazolium cation

$[C_nTHT]^+$, 1-alkyl-tetrahydrothiophene cation

ePC-SAFT, electrolyte Perturbed-Chain Statistical Associating Fluid Theory

γ_i^{eq}, activity coefficient of component i at equilibrium conditions

IL, ionic liquid

Me-PE, 1-(4-methylphenyl)ethanol

Me-ACP, 4-methylacetophenone

NADH, nicotinamide adenine dinucleotide

NAD^+, oxidized form of NADH

$[NTf_2]^-$, bis(trifluoromethanesulfonyl)imide anion

1-PE, 1-phenylethanol

THT, tetrahydrothiophene

UV/Vis, Ultraviolet–visible spectroscopy

SYNTHESES OF TETRAHYDROTHIOPHENE-ILS

The samples of the THT-ILs (1-alkyl-tetrahydrothiophene bis(trifluoromethanesulfonyl)imide, $[C_nTHT][NTf_2]$ (n = 4 – 8)) were prepared according to literature procedures.[1] The ILs were synthesized in two steps. The first step was the alkylation of tetrahydrothiophene, THT with the *n*-iodoalkane, $C_nH_{2n+1}I$ to yield $[C_nTHT]I$. The $[NTf_2]$-ILs were then prepared by an anion exchange with $Li[NTf_2]$ in an aqueous solution (Scheme S1). The anion purity of the THT-ILs was measured by ion chromatography. The purities are given in Table S1.

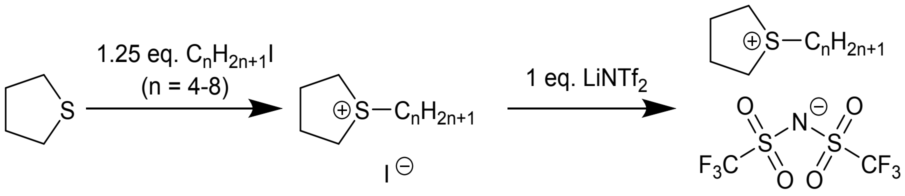

Scheme S1. Synthesis of the tetrahydrothiophene-based ionic liquids.

Table S1. Purity of $[C_nTHT][NTf_2]$ ILs measured by ion chromatography.[a]

IL	anion purity, wt-%
$[C_4THT][NTf_2]$	99.5
$[C_5THT][NTf_2]$	99.5
$[C_6THT][NTf_2]$	99.7
$[C_7THT][NTf_2]$	99.7
$[C_8THT][NTf_2]$	99.6

[a] Sample were prepared by dissolving a defined mass of IL in a defined volume of eluent.

The UV-Vis spectra of the THT-ILs are shown in Figure S1. No absorption was detected for the wavelength used to track the ongoing ADH reaction ($at\ \lambda = 340\ nm$).

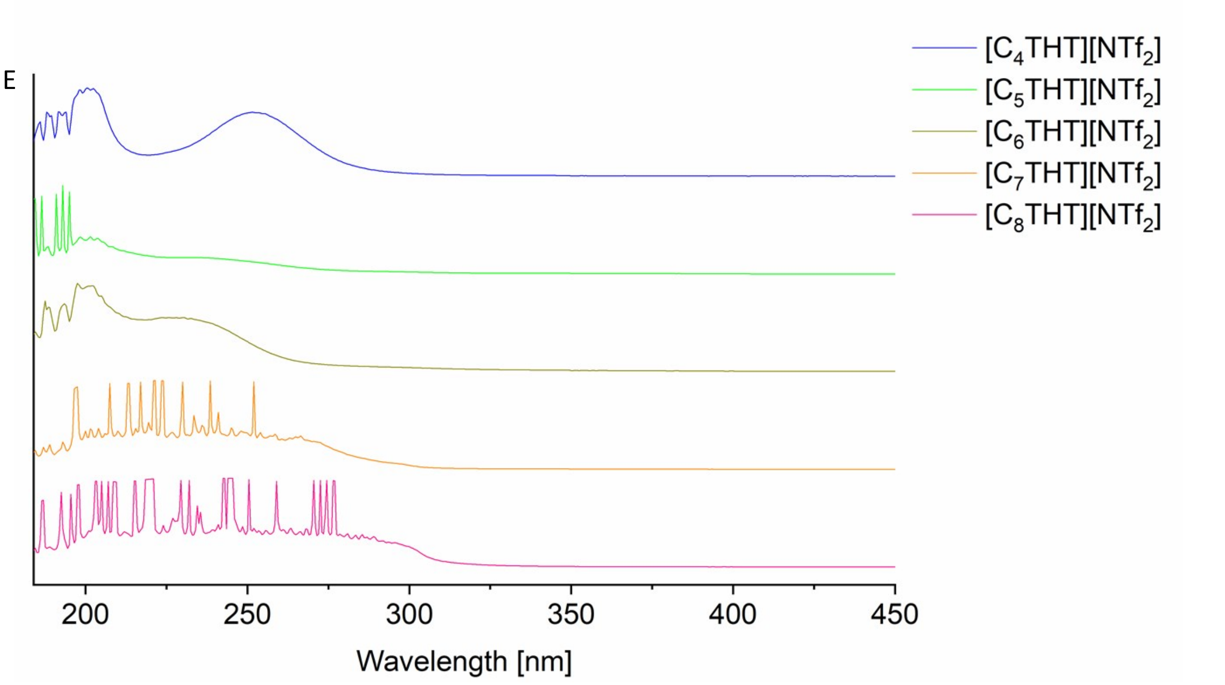

Figure S1. UV-vis spectra of the pure THT-ILs as extinction vs. wavelength.

THERMODYNAMICS OF REACTION EQUILIBRIA

The general background on thermodynamic equilibria is based on the Gibbs free energy of a reacting system $\Delta^R g$ according to equation (1). The relationship between the free energy and the chemical potential μ_i in terms of a thermodynamic chemical equilibrium constant K_{th} has been discussed in detail numerously before. Equation (2) links this relationship to the activity a_i of the reactants and to K_{th},

$$\Delta^R g = \sum_i \nu_i \mu_i \qquad (1)$$

$$K_{th} = K_x \cdot K_\gamma = \prod_i a_i^{\nu_i} = \prod_i (x_i \cdot \gamma_i)^{\nu_i} \qquad (2)$$

where K_γ and K_x are the activity and the mole fraction-based equilibrium constants, respectively, x_i is the mole fraction of reactant i, γ_i the corresponding activity coefficient, and ν_i the stoichiometric factor of component i taking part in the current

reaction. K_{th} only depends on temperature. It has to be noted that the thermodynamic equilibrium constant as used in this work includes the activity of H^+ (equation (3)) due to the application of a Hepes buffer and thus a constant pH-value leading to a constant activity of H^+.

$$K_{th} = K_{th}^{'} \cdot a_{H+} \tag{3}$$

As a benchmark for the prediction of the influence of the THT-ILs with ePC-SAFT, the conversion related to the substrate Me-ACP was used as described in equation (4).

$$X_{Me-ACP} = \frac{x_{0,Me-ACP} - x_{Me-ACP}^{eq}}{x_{0,Me-ACP}} \tag{4}$$

EPC-SAFT MODELLING

To calculate the activity coefficients for modelling the THT-IL influence on conversion of the ADH reaction, and thus K_{γ}, the ePC-SAFT equation of state is used. The activity coefficients were determined according to equation (5) and are based on the standard state 'pure component':

$$\gamma_i(p,T,x) = \frac{\varphi_i(p,T,x_i)}{\varphi_{0,i}(x_i = 1)} \tag{5}$$

Both equations are based on the fugacity coefficient φ, which is calculated with equation (6), where Z is the compressibility factor and μ_i^{res} is the chemical potential.

$$\ln \varphi_i = \frac{\mu_i^{res}(T,v)}{kT} - \ln Z \tag{6}$$

The chemical potential, in turn, is calculated from the residual Helmholtz energy a^{res} (equation (7)), which is a sum of four contributions. The first three contributions account for uncharged components; the hard-chain, dispersion and association terms. Charged species additionally need the fourth term a^{ion}, which for ePC-SAFT[2] is based on the Debye-Hückel theory. In contrast to prior works on enzyme-catalyzed reactions, the term is varied and a concentration-dependent dielectric constant is incorporated according to equation (8). A detailed view on the new approach can be found in the literature.[3]

$$a^{res} = a^{hc} + a^{disp} + a^{assoc} + a^{ion} \tag{7}$$

$$a^{ion} = -\frac{\kappa(\varepsilon(x))}{12\pi k_B T \cdot \varepsilon(x)} \sum_j q_j^2 \cdot x_j \cdot \chi_j(\varepsilon(x)) \tag{8}$$

To calculate the residual Helmholtz energy, ePC-SAFT requires up to five physically meaningful pure-component parameters for each component. These parameters are the number of segments m_i^{seg}, the segment-diameter σ_i, the dispersion-energy parameter between two segments u_i/k_B, k_B being the Boltzmann constant, and the association-energy parameter ε^{AiBi}/k_B and the association-volume parameter k^{AiBi} for an associating component with N association sites. To consider mixtures for binary pairs i and j, combining rules from Berthelot and Lorentz are applied as shown in equations (9) and (10).

$$\sigma_{ij} = \frac{1}{2}(\sigma_i + \sigma_j) \tag{9}$$

$$u_{ij} = \sqrt{u_i u_j}(1 - k_{ij}) \tag{10}$$

For the dispersion energy, k_{ij} is a binary interaction parameter correcting the energy between the pairs, giving a possibility to correlate the ePC-SAFT results to experimental data where necessary.

PARAMETER ESTIMATION FOR THT-ILS

For the parametrization of the THT-ILs, no association is assumed and the IL-ions are modelled individually. Thus, IL-ions are limited to three pure-component parameters. THT-ILs are a new kind of ILs and their IL-cations, in contrast to the $[NTf_2]^-$ -anion, have not yet been parametrized. Due to the very low vapor pressure of ILs, only their pure liquid density at different temperatures is used for the fitting process. Minimizing the objective function (equation (11)) with a Levenberg-Marquardt algorithm, the pure-component parameters for $[C_4THT]^+$ and $[C_8THT]^+$ have been fitted. The pure-component parameters for the intermediate IL-cations $[C_nTHT]^+$ (n = 5, 6, 7) have then been linearly correlated, following the procedure already used in earlier publications.[4,5] The fitting was successful when the deviation to experimental data, also for the intermediate IL-cations, was minimal. The pure-component parameters for the IL-cations, as well as for the other reacting agents and solvents, are listed in Table S2.

$$OF_\rho = \sum_{j=1}^{NP} \left| 1 - \left(\frac{\rho_i^{mod}}{\rho_i^{exp}} \right)_j \right| \tag{11}$$

PREDICTING THT-IL INFLUENCE ON REACTION EQUILIBRIUM

The effect of IL-additives on reaction equilibrium is predictively available through the thermodynamic equilibrium constant K_{th} as described above. The predication is possible by minimizing the objective function described in equation (12) in an iterative process. In an iteration step i, the equilibrium constant $K_{x,i}$ is calculated from variation of the NADH concentration and mass balance. Thereafter, ePC-SAFT is used to calculate $K_{\gamma,i}$. The steps are repeated until the criterion for the objective function is met.

$$OF_x = \sum_{i=1}^{NP} \left| K_{th} - K_{x,i} \cdot K_{\gamma,i} \right| \tag{12}$$

PURE-COMPONENT PARAMETERS FOR EPC-SAFT

Pure-component parameters for the THT-ILs have been fitted to liquid density data measured in this work (see Table S4). The approach used in this work is similar to prior work[4]. Thus only the ILs $[C_4THT][NTf_2]$ and $[C_8THT][NTf_2]$ have been used for fitting and the other IL-cations' pure-component parameters have been interpolated with respect to the molar weight of the respective IL-cation. IL-cation pure-component parameters for $[C_nTHT]$-ILs are calculated based on the equations (13) to (15). The benefit of this approach is that only few experimental data are needed to actually adjust pure-component parameters for a wide number of ILs. However, to stress the approach, linearly interpolated pure-component parameters for the IL $[C_6THT][NTf_2]$ were used to predict its liquid density. The difference between calculated and measured density was found to be less than 1%.

$$m_i^{seg} = 2.227 \cdot 10^{-2} \cdot M_{w,IL-cation\,i} - 1.411 \tag{13}$$

$$\sigma_i = 3.264 \cdot 10^{-3} \cdot M_{w,IL-cation\,i} + 3.810 \tag{14}$$

$$u_i/k_B = 8.028 \cdot 10^{-1} \cdot M_{w,IL-cation\,i} + 741.43 \tag{15}$$

It should be noted that these correlations are only valid until a maximum value of about $M_{w,IL-cation}$ = 200 g/mol as the dispersion energy must not approach infinity but a constant value for ILs with very high cation chain length.

Table S2. ePC-SAFT pure-component parameters for components used in the present work. Segment number m_i^{seg}, segment diameter σ_i, dispersion-energy parameter u_i/k_B, association-energy parameter ε^{AiBi}, association-volume parameter κ^{AiBi}, association sites N_i^{assoc} (donor:acceptor), and valence z.

Component	m_i^{seg} [-]	σ_i [Å]	u_i/k_B [K]	ε^{AiBi} [K]	κ^{AiBi} [-]	N_i^{assoc} [-]	z_i [-]	Ref
Water	1.204	a	353.95	2425.7	0.045	1:1	0	[6]
NADH	27.27	2.2200	260.72	3581.9	0.001	8:8	0	[7]
NAD^+	24.93	2.2300	299.04	3557.3	0.001	8:8	0	[7]
Me-ACP	3.067	3.8273	334.61	0	0.045	1:1	0	b
Me-PE	3.665	3.6651	257.38	2932.0	0.038	1:1	0	b
$[C_4THT]^+$	1.824	4.2844	858.07	-	-	-	+1	b
$[C_5THT]^+$	2.137	4.3302	869.33	-	-	-	+1	b
$[C_6THT]^+$	2.450	4.3760	880.59	-	-	-	+1	b
$[C_7THT]^+$	2.776	4.4218	891.85	-	-	-	+1	b
$[C_8THT]^+$	3.075	4.4675	903.10	-	-	-	+1	b
$[NTf_2]^-$	6.010	3.7469	375.65	-	-	-	-1	b

$a: \sigma_i = 2.7927 + 10.11 \cdot \exp(-0.01775 \cdot T[K]) - 1.417 \cdot \exp(-0.01146 \cdot T[K])$; b this work

Table S3. Binary interaction parameters k_{ij} between water and reactants. Parameters have been fitted to experimental data, independent of the ADH reaction.

Binary system	k_{ij} [-]	Reference
Water - NADH	-0.0585	[7]
Water - NAD^+	-0.0736	[7]
Water - Me-ACP	0.0100	This work
Water - Me-PE	0.0700	This work
Water - $[NTf_2]^+$	0.1100	[3]
Me-PE - $[C_4THT]^+$	-0.0510	This work
Me-PE - $[C_5THT]^+$	-0.1695	This work
Me-PE - $[C_6THT]^+$	-0.0413	This work
Me-PE - $[C_7THT]^+$	-0.2616	This work
Me-PE - $[C_8THT]^+$	-0.08021	This work

DENSITY MEASUREMENTS FOR THT-ILS

IL pure-component parameters have been fitted to pure liquid density data. The density measurements were performed in a u-tube densimeter DMA 4200 (*Anton Paar* GmbH, Graz, Austria) at varying temperatures from 288.15 K to 313.15 K and ambient pressure. The apparatus' uncertainty is $\pm 1.5 \cdot 10^{-6}$ g/cm³. The experimental density data for $[C_nTHT][NTf_2]$ (n = 4, 6, 8) can be found in Table S4. The resulting pure-component parameters are summarized in Table S2.

Table S4. Pure liquid density of the $[C_nTHT][NTf_2]$ (n = 4, 6, 8) ILs at 1 bar.

T [K]	288.15	293.15	298.15	303.15	308.15	313.15
$[C_4THT][NTf_2]$	1465.4	1460.7	1455.9	1451.1	1446.5	1441.7
$[C_6THT][NTf_2]$	1398.6	1394	1389.4	1384.9	1380.4	1375.9
$[C_8THT][NTf_2]$	1345.1	1340.7	1336.3	1332	1327.5	1323.2

REACTION-EQUILIBRIUM MEASUREMENTS

SOLUTION PREPARATION

Solutions were prepared gravimetrically using the analytical scale XS205 DualRange (*Mettler Toledo*, uncertainty 10^{-4} g). Millipore water with Hepes buffer (0.1 mmol/kg) was used as an aqueous basis for the ADH reaction, setting the pH-value to pH = 7 and periodically controlled via a pH-meter (*Mettler Toledo*, accuracy 10^{-2}). The substrates and the IL-additives, according to the required concentration under investigation (Table S5), were placed in 1.5 mL Eppendorf vials and mixed with the aqueous solution. The vials were then placed in a ThermoMixer (*Eppendorf*) at constant reaction conditions (1000 rpm and 298 K). Afterwards, the enzyme ADH was solved and the vial was left in the ThermoMixer for the reaction time.

Table S5: Concentration of the prepared substrates before reaction and exact concentration of the respective THT-IL for the ADH reaction in mmol/L buffer and the uncertainty stemming from balancing.

Component	$[C_4THT][NTf_2]$		$[C_5THT][NTf_2]$		$[C_6THT][NTf_2]$		$[C_7THT][NTf_2]$		$[C_8THT][NTf_2]$	
NADH	0.503	±0.004	0.492	±0.001	0.497	±0.001	0.503	±0.002	0.508	±0.001
Me-ACP	0.510	±0.004	0.468	±0.001	0.481	±0.001	0.503	±0.001	0.557	±0.001
THT-IL	9.90	±0.05	11.35	±0.02	10.26	±0.02	10.25	±0.01	9.68	±0.13

REACTION-EQUILIBRIUM MEASUREMENTS

Reaction-equilibrium measurements for the ADH reaction with reactant Me-ACP was carried out by two different methods. First, a prepared solution was measured continuously in an *Eppendorf* Biospectrometer. Therefore, the stock solution was filled in a Cuvette SUPRASIL TYP 114-QS from *Hellma Analystics*. The enzyme was added, and the solution was carefully stirred (with 800 rpm) to ensure good mixing. Then, the cuvette was placed in the Biospectrometer (tempered to 298 K) and the extinction of NADH was measured at a constant wavelength of 340 nm. With ongoing reaction, the extinction of NADH reduces with the production of NAD^+, thus giving an easily quantifiable approach. The other reactants and the THT-ILs experience no absorption at this wavelength. Second, duplicate measurements were performed in a ThermoMixer (*Eppendorf*) as described above. After the reaction equilibrium was reached, the equilibrium concentration of NADH was measured in the Biospectrometer. In total, the influence of THT-ILs on the ADH reaction was tested three times for every IL and concentration. An extinction over time plots for the reaction is exemplarily shown in Figure S2 for the neat reaction in buffer. All reactant concentrations in equilibrium are available through mass balances according to equations (16) to (18). All concentrations are measured in molarities c [mol/L buffer].

$$\Delta c_{NADH} = c_{NADH}^{0} - c_{NADH}^{eq} \quad (16)$$

$$c_{Me-ACP}^{eq} = c_{Me-ACP}^{0} - c_{NADH}^{eq} \quad (17)$$

$$c_{NAD+}^{eq} = \Delta c_{NADH} = c_{Me-PE}^{eq} \quad (18)$$

VERIFYING REACTION EQUILIBRIA

To state the reach of real reaction equilibrium, two effects have to be discussed and validated. First, there is the possibility of a deactivated enzyme and second, a limitation of the substrate concentration. Both effects lead to a constant extinction level of NADH while the reaction is observed in the Biospectrometer, although the reaction equilibrium was not reached. A deactivation of the enzyme can be tested straight forward by adding new stock solution with solved, active enzyme. If the reaction equilibrium was reached, no further decrease in NADH extinction is observed, except for the extinction due to the addition of stock solution. For the second possibility, substrate should be added accordingly. Since NADH is a substrate itself, the NADH extinction initially increases but eventually reaches about the same value as before the substrate addition. Results for the ADH reactions for the neat reaction in aqueous buffer solution and with the THT-IL additives are shown in Table S6.

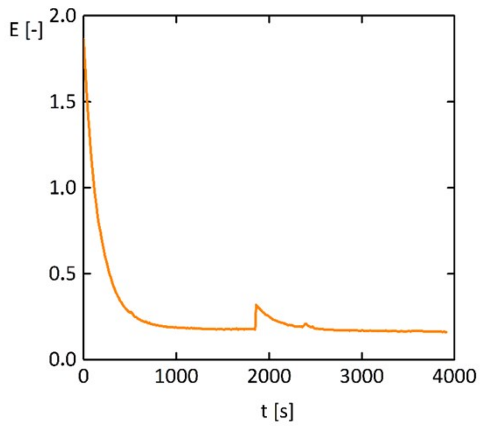

Figure S2. Extinction E over time plot for the ADH reaction in aqueous buffer (neat) at 298 K and at λ = 340 nm. Verification of equilibrium by addition of new substrate after 1800 s and subsequent reduction of the extinction to the value before substrate addition.

Table S6. Equilibrium conversion for the ADH reaction at 298 K for the neat reaction and for the reaction with 10 mmol/kg THT-ILs as additives.

Medium	conversion	±	uncertainty
Neat buffer	0.86948	±	0.02614
10 mmol/L [C_4THT] IL	0.80396	±	0.03906
10 mmol/L [C_5THT] IL	0.92392	±	0.07047
10 mmol/L [C_6THT] IL	0.81546	±	0.04616
10 mmol/L [C_7THT] IL	0.96378	±	0.00152
10 mmol/L [C_8THT] IL	0.81243	±	0.00570

MEASURING THE CRITICAL MICELLE CONCENTRATION

For the investigation of the odd-even effect and the local surrounding of the THT-ILs, the concentration-dependent surface tensions (σ) of the THT-ILs were measured with a ring tensiometer in order to determine the respective CMCs. For this purpose, a stock solution of the respective THT-IL in water was prepared and a series of dilutions was performed.

Three measurements for each concentration and accordingly the mean values for the CMC was determined. The measurements were also repeated with the addition of the product Me-PE. The results for both systems are shown in Table S7.

Table S7. Dilution series for the measurements without and with product.

IL-concentration c [mol/L]	log(c)	σ without product [mN/m]	σ with product [mN/m]
$[C_4THT][NTf_2]$			
$4.00 \cdot 10^{-2}$	-1.4	44.1	42.3
$3.00 \cdot 10^{-2}$	-1.5	43.5	42.2
$2.00 \cdot 10^{-2}$	-1.7	43.6	43.1
$1.00 \cdot 10^{-2}$	-2.0	49.9	49.1
$5.00 \cdot 10^{-3}$	-2.3	56.4	54.6
$2.50 \cdot 10^{-3}$	-2.6	62.4	60.5
$1.25 \cdot 10^{-3}$	-2.9	67.6	62.3
$6.25 \cdot 10^{-4}$	-3.2	69.9	62.0
$3.13 \cdot 10^{-4}$	-3.5	70.9	62.8
$1.56 \cdot 10^{-4}$	-3.8	70.9	66.3
$[C_5THT][NTf_2]$			
$4.00 \cdot 10^{-2}$	-1.4	44.1	43.3
$3.00 \cdot 10^{-2}$	-1.5	43.5	42.3
$2.00 \cdot 10^{-2}$	-1.7	43.6	42.5
$1.00 \cdot 10^{-2}$	-2.0	49.9	42.5
$5.00 \cdot 10^{-3}$	-2.3	56.4	49.3
$2.50 \cdot 10^{-3}$	-2.6	62.4	54.4
$1.25 \cdot 10^{-3}$	-2.9	67.6	59.1
$6.25 \cdot 10^{-4}$	-3.2	69.9	62.1
$3.13 \cdot 10^{-4}$	-3.5	70.9	65.8
$1.56 \cdot 10^{-4}$	-3.8	70.9	67.2
$[C_6THT][NTf_2]$			
$4.00 \cdot 10^{-2}$	-1.4	43.4	42.1
$3.00 \cdot 10^{-2}$	-1.5	41.9	41.3
$2.00 \cdot 10^{-2}$	-1.7	42.5	41.3
$1.00 \cdot 10^{-2}$	-2.0	42.6	41.8
$5.00 \cdot 10^{-3}$	-2.3	50.0	48.4
$2.50 \cdot 10^{-3}$	-2.6	56.5	54.0
$1.25 \cdot 10^{-3}$	-2.9	62.3	60.1
$6.25 \cdot 10^{-4}$	-3.2	67.8	62.8
$3.13 \cdot 10^{-4}$	-3.5	70.2	65.6
$1.56 \cdot 10^{-4}$	-3.8	70.8	64.3
$[C_7THT][NTf_2]$			
$4.00 \cdot 10^{-2}$	-1.4	42.8	42.0
$3.00 \cdot 10^{-2}$	-1.5	42.8	42.2
$2.00 \cdot 10^{-2}$	-1.7	42.8	42.1
$1.00 \cdot 10^{-2}$	-2.0	42.8	42.2
$5.00 \cdot 10^{-3}$	-2.3	49.4	48.5
$2.50 \cdot 10^{-3}$	-2.6	55.5	54.1
$1.25 \cdot 10^{-3}$	-2.9	60.1	58.1
$6.25 \cdot 10^{-4}$	-3.2	63.4	60.0
$3.13 \cdot 10^{-4}$	-3.5	66.2	61.1
$1.56 \cdot 10^{-4}$	-3.8	66.5	61.5
$[C_8THT][NTf_2]$			
$4.00 \cdot 10^{-2}$	-1.4	42.2	40.5
$3.00 \cdot 10^{-2}$	-1.5	42.6	41.7
$2.00 \cdot 10^{-2}$	-1.7	42.6	41.6
$1.00 \cdot 10^{-2}$	-2.0	43.2	41.7
$5.00 \cdot 10^{-3}$	-2.3	46.5	46.6
$2.50 \cdot 10^{-3}$	-2.6	55.3	53.3
$1.25 \cdot 10^{-3}$	-2.9	59.7	56.2
$6.25 \cdot 10^{-4}$	-3.2	63.0	56.9
$3.13 \cdot 10^{-4}$	-3.5	65.7	58.4
$1.56 \cdot 10^{-4}$	-3.8	67.3	60.8
$7.81 \cdot 10^{-5}$	-4.1	66.4	61.0

For the determination of the CMC, the data are plotted and the two value ranges are linearized. The concentrations of the critical micelle formation are given by the intersection. The CMC is obtained by delogarithmization. An exemplary application can be seen in Figure S3. The results are shown in Table S8.

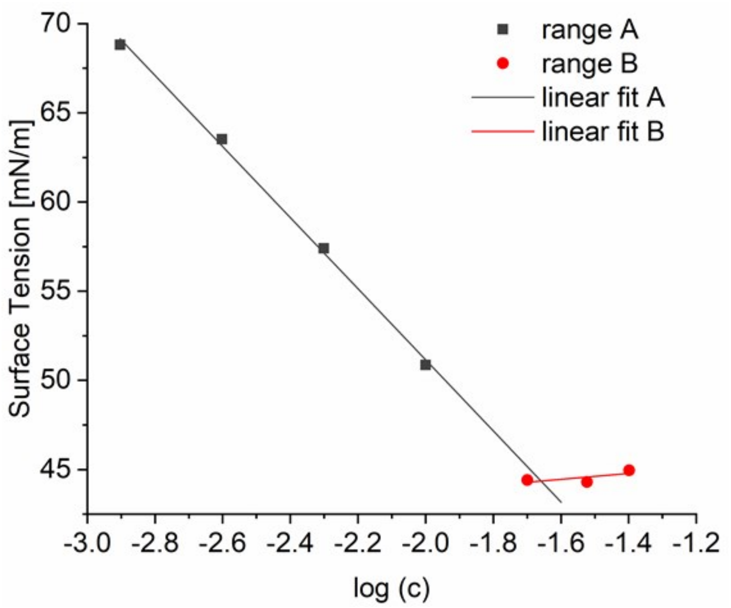

Figure S3. Example application of the measured values and linearization for determination of the intersection (CMC).

Table S8. CMCs with and without product, difference and standard deviation.

IL	CMC without product [a] [mmol/L]	CMC with product [b] [mmol/L]	Difference in the CMC [mmol/L]	Difference in the CMC [%]	Standard deviation for difference in the CMC [%]
[C_4THT][NTf_2]	21.9	20.8	-1.1	-5.2	0.3
[C_5THT][NTf_2]	10.4	14.3	3.9	37.8	0.8
[C_6THT][NTf_2]	12.6	11.0	-1.6	-12.3	0.3
[C_7THT][NTf_2]	12.1	12.9	0.8	6.4	0.3
[C_8THT][NTf_2]	11.9	10.7	-1.2	-10.1	0.1

[a] Pure Millipore water. [b] In 1 mmol Me-PE/kg Millipore water.

ADH REACTION IN NEAT THT-ILS

The investigations described until this point address the effect of THT-ILs at rather low concentrations of about 10 mmol/L es per kg water. The question rises whether the reaction can be realized also in pure IL. Thus, the holistic approach in this article was extended to investigations of enzyme stability in the pure THT-ILs. The application of ILs to bioreactions has been successful for different enzymatic reactions and highly benefitted the conversion. However, the stability of the enzyme in presence of ILs at high concentration is usually a challenge,[8–10] which can be solved only by immobilization of the enzyme.[11] Accordingly, the influence of the pure THT-IL on the enzyme activity was investigated over a time span of 31 days (Figure S4).

Periodic sampling of the ADH solution was performed. Therefore, stock reactant solution was introduced to the reaction media and the reaction progress was monitored by UV-Vis detection of NADH. The results in Figure 4 prove that the reaction proceeds, but the kinetics is decreased compared to the reaction water. This is probably caused by transport limitation in the pure IL compared to water. In addition to the enhancement effect of [C_5THT][NTf_2] on conversion, even after more than one

month, the activity of the ADH was unaltered and the enzyme found to be stable. The difference in extinction peak heights is related to the different amount of reactants (Me-ACP and NADH) added to reaction medium at the specified times. For the activity analysis the reactants were added frankly as no quantification was necessary. For comparison, ADH in an aqueous buffer solution was found to be active only for 2 h at room temperature. In pure [C_5THT][NTf_2], the time span of ADH activity was thus extended about 500 times. Usually, the pure enzyme has to be stored under dry conditions at - 18 °C. Storing it in [C_5THT][NTf_2] thus gives advantages, making it easily dispensable for the reaction, while enhancing the activity and conversion.

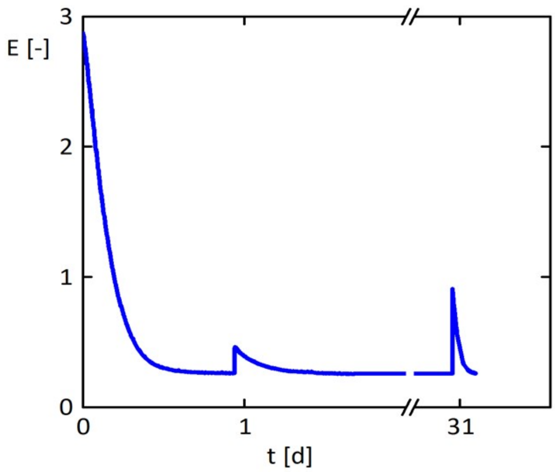

Figure S4. ADH activity (monitored by UV-Vis extinction (E) of NADH at λ = 340 nm over time) towards the investigated synthesis of Me-PE after one day and after 31 days and addition of new reactants NADH and Me-ACP. Reaction reaches equilibrium again within short time. ADH enzyme stored in pure [C_5THT][NTf_2] for 31 days at room temperature.

REFERENCES

(1) Paulsson, H.; Hagfeldt, A.; Kloo, L. Molten and Solid Trialkylsulfonium Iodides and Their Polyiodides as Electrolytes in Dye-Sensitized Nanocrystalline Solar Cells. *J. Phys. Chem. B* **2003**, *107*, 13665–13670.

(2) Held, C.; Reschke, T.; Mohammad, S.; Luza, A.; Sadowski, G. ePC-SAFT revised. *Chem. Eng. Res. Des.* **2014**, *92*, 2884–2897.

(3) Bülow, M.; Ji, X.; Held, C. Incorporating a concentration-dependent dielectric constant into ePC-SAFT. An application to binary mixtures containing ionic liquids. *Fluid Phase Equil.* **2019**, *492*, 26–33.

(4) Ji, X.; Held, C.; Sadowski, G. Modeling imidazolium-based ionic liquids with ePC-SAFT. *Fluid Phase Equil.* **2012**, *335*, 64–73.

(5) Ji, X.; Held, C.; Sadowski, G. Modeling imidazolium-based ionic liquids with ePC-SAFT. Part II. Application to H2S and synthesis-gas components. *Fluid Phase Equil.* **2014**, *363*, 59–65.

(6) Cameretti, L. F.; Sadowski, G. Modeling of aqueous amino acid and polypeptide solutions with PC-SAFT. *Chem. Eng. Process.* **2008**, *47*, 1018–1025.

(7) Wangler, A.; Loll, R.; Greinert, T.; Sadowski, G.; Held, C. Predicting the high concentration co-solvent influence on the reaction equilibria of the ADH-catalyzed reduction of acetophenone. *J. Chem. Thermodynamics* **2019**, *128*, 275–282.

(8) Erbeldinger, M.; Mesiano, A. J.; Russell, A. J. Enzymatic catalysis of formation of Z-aspartame in ionic liquid - An alternative to enzymatic catalysis in organic solvents. *Biotech. Progress* **2000**, *16*, 1129–1131.

(9) Madeira Lau, R.; Sorgedrager, M. J.; Carrea, G.; van Rantwijk, F.; Secundo, F.; Sheldon, R. A. Dissolution of Candida antarctica lipase B in ionic liquids: effects on structure and activity. *Green Chem.* **2004**, *6*, 483–487.

(10) Lozano, P.; Diego, T. de; Guegan, J. P.; Vaultier, M.; Iborra, J. Stabilization of [alpha]-chymotrypsin by ionic liquids in transesterification reactions. *Biotechnol. Bioeng.* **2001**, *75*, 563–569.

(11) Moniruzzaman, M.; Nakashima, K.; Kamiya, N.; Goto, M. Recent advances of enzymatic reactions in ionic liquids. *Biochem. Eng. J.* **2010**, *48*, 295–314.
